# Induced plasticity alters responses to conspecific interactions in seedlings of a perennial grass

**DOI:** 10.1038/s41598-021-93494-0

**Published:** 2021-07-16

**Authors:** Alicia J. Foxx

**Affiliations:** Genomics and Bioinformatics Research Unit, United States Department of Agriculture, Agricultural Research Service, 1600 SW 23rd Drive, Gainesville, FL 32608 USA

**Keywords:** Ecology, Plant sciences

## Abstract

Plants can interact with different individuals in their lifetime which may lead to plastic response that affect performance. If conspecific interactions are altered through previous plastic responses that could affect stabilizing niche mechanisms, in which conspecifics compete more intensely to promote diversity and coexistence. Here, I show interactions between *Pascopyrum smithii* and conspecifics resulted in largely canalized traits, whereas *P. smithii* with an invasive grass, *Bromus tectorum* resulted in plastic responses for root mass (*p* = 0.02), shoot mass (*p* < 0.0001), root mass fraction (*p* = 0.003) and plant height (*p* < 0.0001). A subset of individuals transplanted from these two interaction treatments which were moved with new, same aged conspecifics showed that previous interactions led to differing trait relationships: increases in the number of leaves for the interspecific-induced plants were related to increases in non-focal leaf production, whereas increases in the number of leaves for the intraspecific-induced plants were related to decreases in the non-focal plants (*R*^2^ = 0.52, *p* = 0.006). These results suggest that previous intraspecific interactions intensify conspecific competition and stabilize subsequent interactions with conspecifics by imposing greater competition, and that invasive-interspecific interactions can weaken stabilizing niche mechanisms, thus negatively influencing species coexistence.

## Introduction

Plant plastic responses include morphological, physiological, and behavioral changes that can influence resource capture, fitness, and survival^1–4^. Plants respond plastically to both biotic and abiotic factors, for example, water stress induces greater root length which coincides with greater water uptake^5^ and maize competing with *Sorghum* reduced growth rate and root production in maize^6^. The identity of the interacting neighbor matters; evidence suggests that species in their invasive range induce plastic responses in co-occurring native species, and their presence results in transgenerational and adaptive signals in the native plant^7–10^. Additionally, *Bromus tectorum*, is highly competitive with root system traits primed for rapid resource capture^11,12^ which influenced greater allocation to root mass and fine roots by the native *Poa secunda* following competition^13^, demonstrating the impact invasive species interactions have on plastic responses of native species.

In addition to induced plastic responses, a dearth of evidence shows that biotic-induced plastic responses influence subsequent interactions^14–16^. One example showed that leaf damage that simulated herbivory in clipped sagebrush plants induced production of volatile compounds in nearby tomato plants which reduced herbivory for the tomato plants^17^. Plants, particularly perennial plants, can interact with different plant species in their lifetime due to immigration, exposure to disturbance, or changes in native and invasive species cover, that can influence subsequent interactions. Additionally, native species out planted in restoration settings may experience interactions in nursery settings that could affect plant performance when out planted. Alexander et al.^18^ transplanted plant communities to a warmer climate and showed that this resulted in intensified competition with new competitors. While this provides support that plant-plant interactions can change when meeting new neighbors, most plant studies that evaluate the impact of biotic-induced plasticity involve impacts from organisms of another trophic level (e.g., insects^17^). This reveals a knowledge gap in our understanding of plant-plant induced changes to subsequent plant interactions.

Importantly, there are key relationships that can have ramifications for plant communities if altered by induced plasticity. Specifically, the strength and direction of conspecific interactions are important to species coexistence for stabilizing niche mechanisms^19,20^, in which population self-limiting mechanisms important to coexistence and diversity are supported by negative conspecific interactions. Therefore, changes to conspecific interactions have the potential to affect stabilizing mechanisms and species coexistence. Furthermore, fitness inequalities^19–21^ are also important to species coexistence and they are trait differences that lead to inferior and superior competitors, or a trait hierarchy, that leads to competitive exclusion. But a small number of studies demonstrate the importance of plasticity on species coexistence: Muthukrishnan et al.^22^ showed via simulations under competition-colonization trade-off-based species coexistence mechanism, that plasticity broadened the number of species, environments, and competitive contexts that resulted in coexistence. Plasticity also helped to maintain competitive ability across different water levels and strengthened stabilizing differences of some traits^23^. Generally, plasticity can contribute to complementary resource capture and trait differentiation in communities that may contribute to niche differentiation^24,25^, and thus plasticity is crucial to consider to advance our understanding of plasticity effects on species coexistence and community dynamics. This can be achieved by testing the impacts of previous interactions on subsequent interactions via studies that directly manipulate plasticity and quantify the effects on interactions^26^.

To address these knowledge gaps on plant-plant induced changes and whether this can affect conspecific interactions, I empirically determine whether intraspecific or interspecific interactions induce plastic responses, and whether these induced responses impact subsequent intraspecific interactions–interactions important to species coexistence. I performed transplant interaction experiments in a greenhouse and used above- and belowground traits and morphology to quantify plasticity and interaction outcomes. I used a grass native to the Western United States, *Pascopyrum smithii*, and the co-occurring invasive grass, *Bromus tectorum*, which has been shown to induce plastic responses in native grasses^13^. Then I assessed whether plasticity was induced and whether it led to differing subsequent intraspecific interactions that underlie stabilizing niche mechanisms. I hypothesized that (1) intraspecific and interspecific interactions will induce plastic responses in *P. smithii*, (2) intraspecific-induced and interspecific-induced focal *P. smithii* plants will differ in traits and morphology with new conspecifics, and (3) intraspecific-induced and interspecific-induced *P. smithii* will differently affect new conspecific neighbors.

## Materials and methods

### Study materials and process

**Figure 1:**
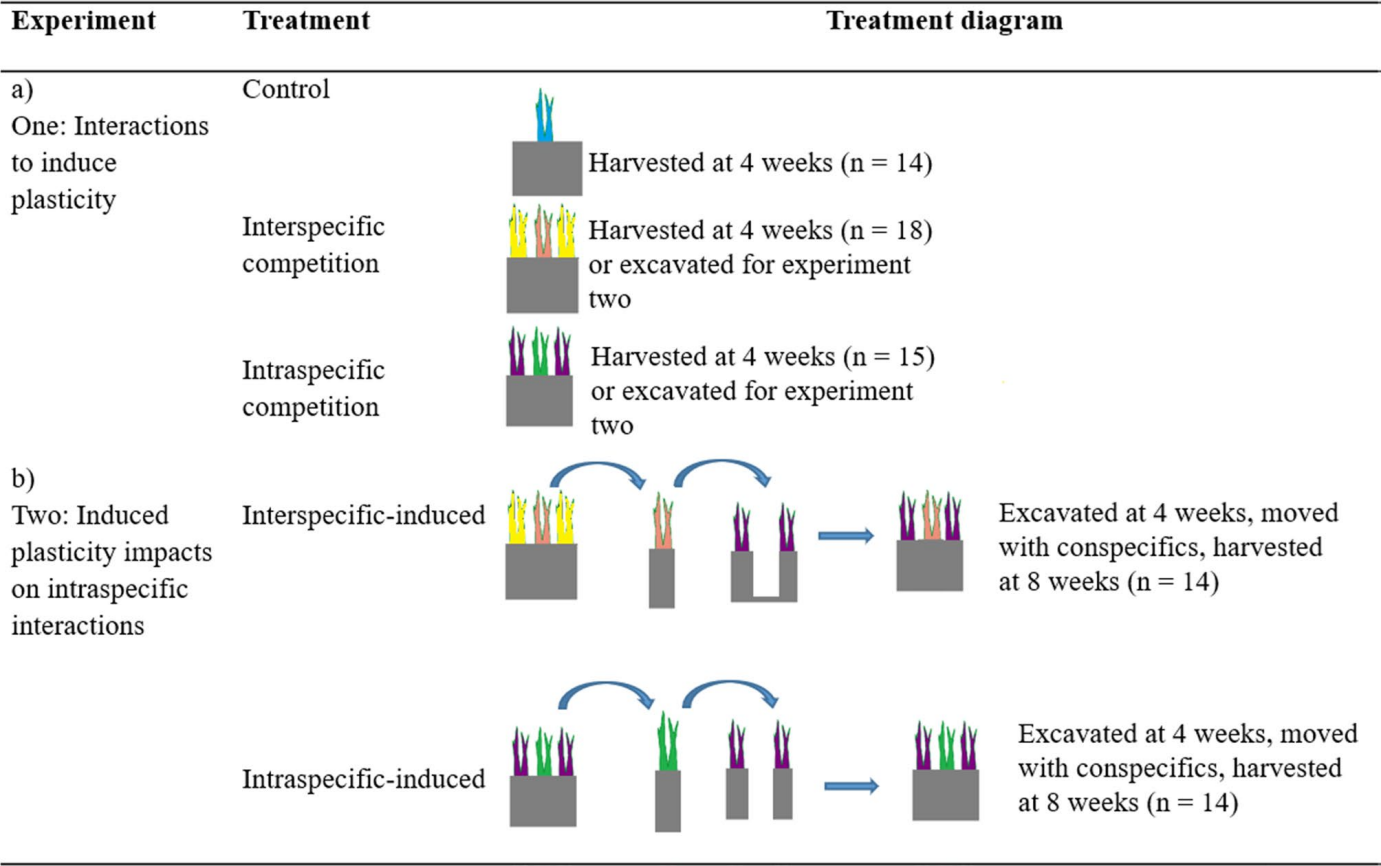
Interaction treatments and final sample size for the greenhouse experiments with *Pascopyrum smithii* focal plants (green, pink). Interspecific interaction treatments are with *Bromus tectorum* (purple) neighbors. (**a**) Experiment one using interactions to induce plasticity in the focal plant, (**b**) experiment two on the consequences of induced plasticity. Treatment sample sizes are in parentheses.

I conducted two experiments in a greenhouse (Fig. 1, see Supplementary Fig S1) between June 2017 and September 2017 at the Chicago Botanic Garden (Glencoe, IL, USA) with the goal of inducing responses and using transplants to evaluate the effects of plastic responses on subsequent interactions. Specifically, the goal of the first experiment was to induce plastic responses in *Pascopyrum smithii* (Rydb.) Á. Löve cv. ‘Arriba’ through plant-plant interactions with conspecific or heterospecific neighbors (*Bromus tectorum* L.). The goal of the second experiment was to assess if transplanted intraspecific-induced and interspecific-induced *P. smithii* from the first experiment differed in competitive outcomes with new conspecific neighbors.

The focal plant *P. smithii* is a perennial rhizomatous bunchgrass native to the western United States and the non-focal plant is *B. tectorum*, a non-native invasive annual grass species. Seeds of *P. smithii* were purchased in July 2014 (Central Milling Wheatland, UT, USA), and *B. tectorum* seeds were wild collected from Grand County, UT (38.998°N, − 110.177°W) in June 2013. Seed collection was permitted by the director of the Bonderman Field Station at Rio Mesa and research performed conformed to the Chicago Botanic Garden Ethics Code.

I germinated 500 seeds (germination determined via radicle emergence). Germination timing varied, but I planted all interacting germinants with similar sized emerged radicles to each pot simultaneously to minimize size differences.

Experiments one and two ran from 6 Jun. 2017 to 18 Sept. 2017 with an average of 21 °C/ 16 °C day/night with lighting supplementing ambient lighting from outdoors. I used 20—7.6 cm × 7.6 cm × 15.2 cm rectangular pots (Stuewe & Sons, OR, USA) per treatment for each experiment with fine commercial sand. I watered plants three times weekly, rotated trays weekly, and added Murashige–Skoog (Sigma Aldrich, MO, USA) half strength nutrients (4 g/L) at week two of both experiments.

### Experiment one: interactions to induce plasticity

The treatments applied were intraspecific interactions with three interacting *P. smithii* seedlings in each pot (n = 15); interspecific interactions with one *P. smithii* seedling surrounded by two *B. tectorum* seedlings in each pot (n = 18); and one *P. smithii* growing alone (n = 14) serving as the control group (Fig. 1a). Plants were evaluated at the seedling stage as seedlings are more sensitive to competition^27,28^ and can exhibit plastic responses in above- and belowground traits^29^, thus plastic responses can be induced at an early life stage. After 4 weeks of growth, plants were randomly designated to either be harvested for data collection or transplanted to new pots with two *P. smithii* neighbors for experiment two.

At harvest, I gently separated the plants at the root systems under water. Next, I measured the plant height then scanned each focal plant using an Epson expression 10000XL scanner (Epson, CA, USA) and counted the number of leaves and root tips from the scanned images. I recorded root and shoot mass after drying in an oven at 95 °C for 5 days and calculated the functional trait root mass fraction (RMF: root mass/ plant mass) (data;^30^).

### Experiment two: induced plasticity impacts on intraspecific interactions

Focal plants were transplanted using spatulas from the pots used in the first study after watering the pots and minimizing focal plant root disturbance (Fig. 1b, Supplementary Figure S1). I transplanted focal seedlings to randomized pots with two *P. smithii* plants of the same age (4 weeks old). *Pascopyrum smithii* plants in the recipient pots were propagated and grown using the same procedure as experiment one. This resulted in two treatments: (1) intraspecific-induced plants transplanted with two same-age intraspecific plants (n = 14), and (2) interspecific-induced plants transplanted with two same-age intraspecific plants (n = 14) (Fig. 1b), and an undisturbed control treatment in which one *P. smithii* grew alone for 8 weeks (n = 12; not used for analytical comparisons as the plants were not transplanted or disturbed as in the treatments). I washed plants of sand after harvesting and I counted the cumulative number of rhizomes of all three plants in each pot because the root systems were highly tangled together and with mesh used to keep the sand in place. I scanned the plants then counted the number of leaves for the focal and non-focal plants and dried plants in a drying oven at 95 °C for 5 days prior to weighing focal plant aboveground biomass.

### Statistical analyses

All analyses were performed in R version 4.0.2 as well as figures^31^. I applied a square root transformation to shoot mass and RMF data to meet assumptions of normality for experiment one. In experiment one, to test whether plant morphology differs between the control group and both intraspecific and interspecific interaction treatments, I contrasted treatment means of the focal plant with the control group to assess if plasticity was induced. I used linear models with treatment as a categorical predictor variable, and shoot mass, plant height, root mass, and RMF as response variables in separate models. I used a generalized linear model with a Poisson error distribution as the count data distribution^32^ to analyze the effects of treatment on the number of root tips and number of leaves. I used stepwise backwards elimination of nonsignificant variables (*p*  > 0.05) to select the minimally adequate model for the linear and generalized linear mixed effects models^32^, comparing the model with the interaction treatment variable to the null model.

In experiment two, to test whether plant morphology differs between intraspecific-induced and interspecific-induced plants following new intraspecific interactions, I contrasted treatment means of the intraspecific-induced and interspecific-induced treatments. I used a linear model with treatment as a categorical predictor variable and focal plant shoot mass as the response variable. I used a generalized linear model with a Poisson error distribution to analyze the effects of treatment on cumulative rhizome count and number of leaves of the focal plant. I used stepwise backwards elimination of nonsignificant variables (*p* >  0.05) to select the minimally adequate model for the linear and generalized linear mixed effects models^32^, comparing the model with the interaction treatment variable to the null model. To test whether intraspecific-induced and interspecific-induced individuals imposed competition differently on new conspecifics, I calculated trait hierarchy for leaf count between the focal plant and non-focal plant with a modification on trait hierarchy as in Kraft et al.^33^ for two neighbors as in Eq. (1):

1 $$a - avg\left( {b_{1} ,~b_{2} } \right)$$

where *a* is the number of leaves for focal plant, and the average of the number of leaves for non-focal plants b_1_ and b_2_. I used a linear model to compare leaf trait hierarchy by treatment and a generalized linear model with continuous predictor of focal plant number of leaves and a response of the average number of leaves produced by the non-focal neighbors with treatment (intraspecific-induced and interspecific-induced) as a categorical variable.

## Results

**Figure 2:**
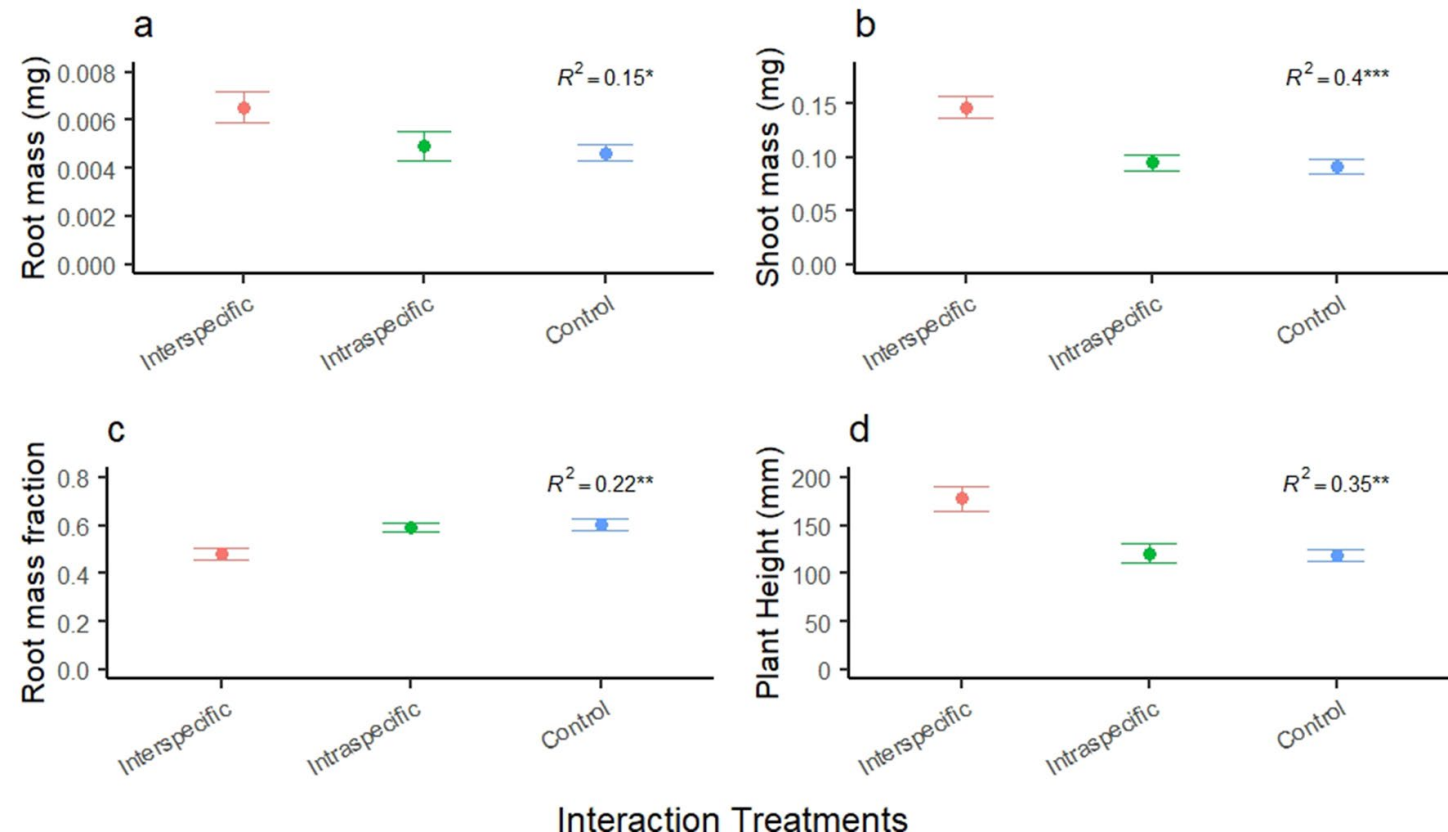
Mean ± standard error of the treatment groups for *Pascopyrum smithii* plants growing alone, and with intraspecific and interspecific neighbors for (**a**) shoot mass, (**b**) root mass, (**c**) RMF (root mass fraction), and (**d**) plant height. Significance levels are denoted by ns *P* > 0.5; *P ≤ 0.05; **P ≤ 0.01; ***P ≤ 0.001.

Plant morphology differed between the control group and treatment groups for some measures when intraspecific neighbors (*P. smithii* conspecifics) and interspecific neighbors (*P. smithii* and *B. tectorum*) interacted. Root mass, shoot mass, and plant height were greatest in the interspecific treatment and similar between the control and intraspecific treatment, however RMF was lowest in the interspecific treatment and similar between the control and intraspecific treatment, and root tip count and number of leaves did not differ by treatment. Root mass contrast marginally differed by treatment compared to the control (*R*^2^ = 0.15, *F*_2,36_ = 3.15, *p* = 0.055, Fig. 2a) and between the control and interspecific interaction (*p* = 0.02), but not for the intraspecific interaction (*p* = 0.4). Shoot mass contrast differed by treatment compared to the control (*R*^2^ = 0.4, *F*_2,36_ = 12.14, *p* < 0.0001, Fig. 2b) and between the control group for intraspecific interaction (*p* = 0.04) and the interspecific interaction (*p* < 0.0001). Root mass fraction differed by treatment compared to the control (*R*^2^ = 0.22, *F*_2,36_ = 5.1, *p* = 0.01, Fig. 1c) and between the control and the interspecific (*p* = 0.003), but not for the intraspecific interaction (*p* = 0.2). Plant height differed by treatment compared to the control (*R*^2^ = 0.35, *F*_2,36_ = 9.3, *p* = 0.0006, Fig. 1d) and between the control and the interspecific (*p* < 0.0001) and the intraspecific interaction (*p* = 0.05). Root tip count and number of leaves did not differ by treatment compared to the control (*p* = 0.9 and *p* = 0.8, respectively^30^).

**Figure 3:**
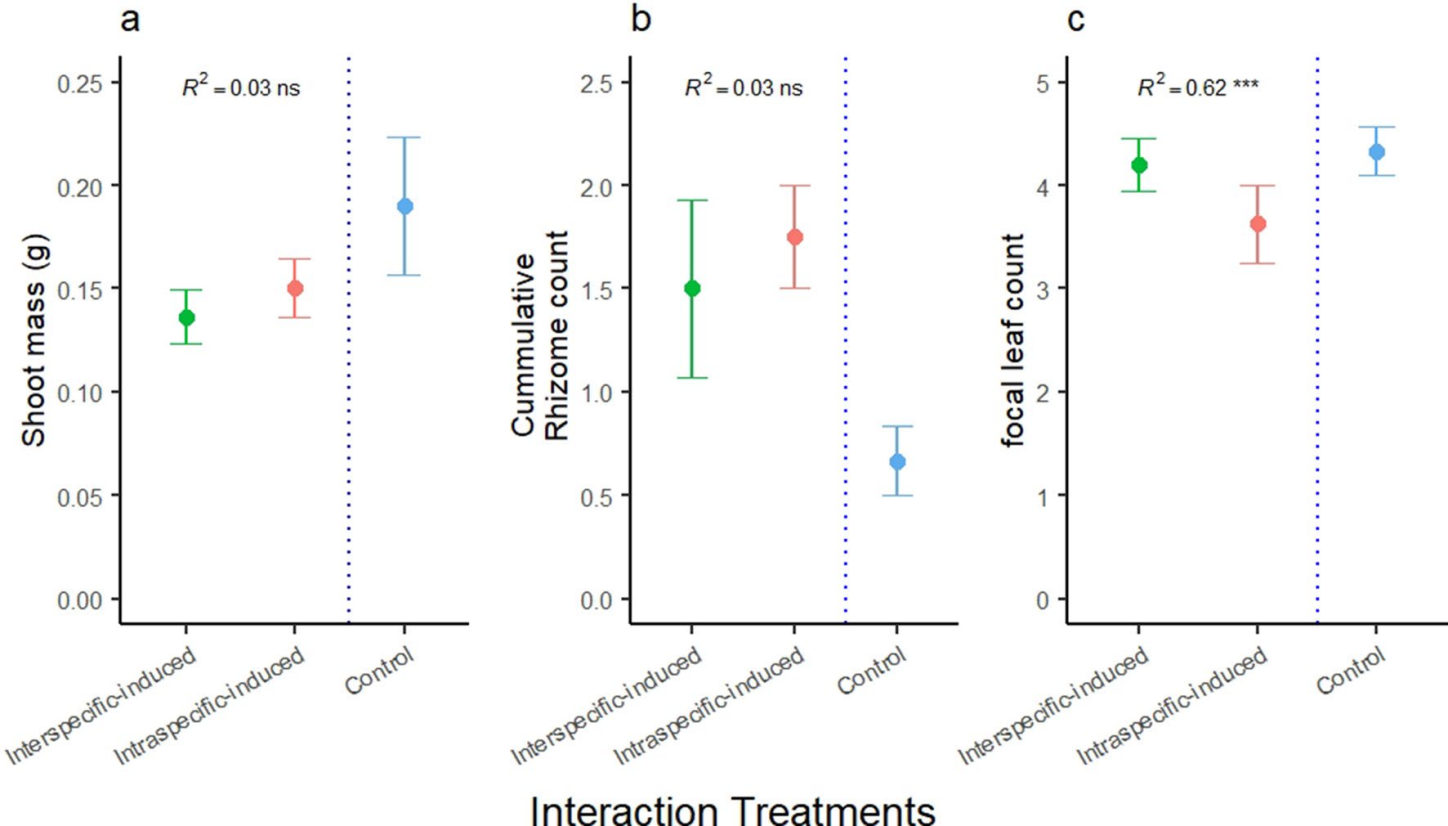
Mean ± standard error of *Pascopyrum smithii* plants induced by intraspecific and interspecific neighbors for (**a**) focal plant shoot mass, (**b**) cumulative rhizomes per pot, and (**c**) focal plant number of leaves. Significance levels are denoted by ns *P* > 0.5; *P ≤ 0.05; **P ≤ 0.01; ***P ≤ 0.001.

**Figure 4:**
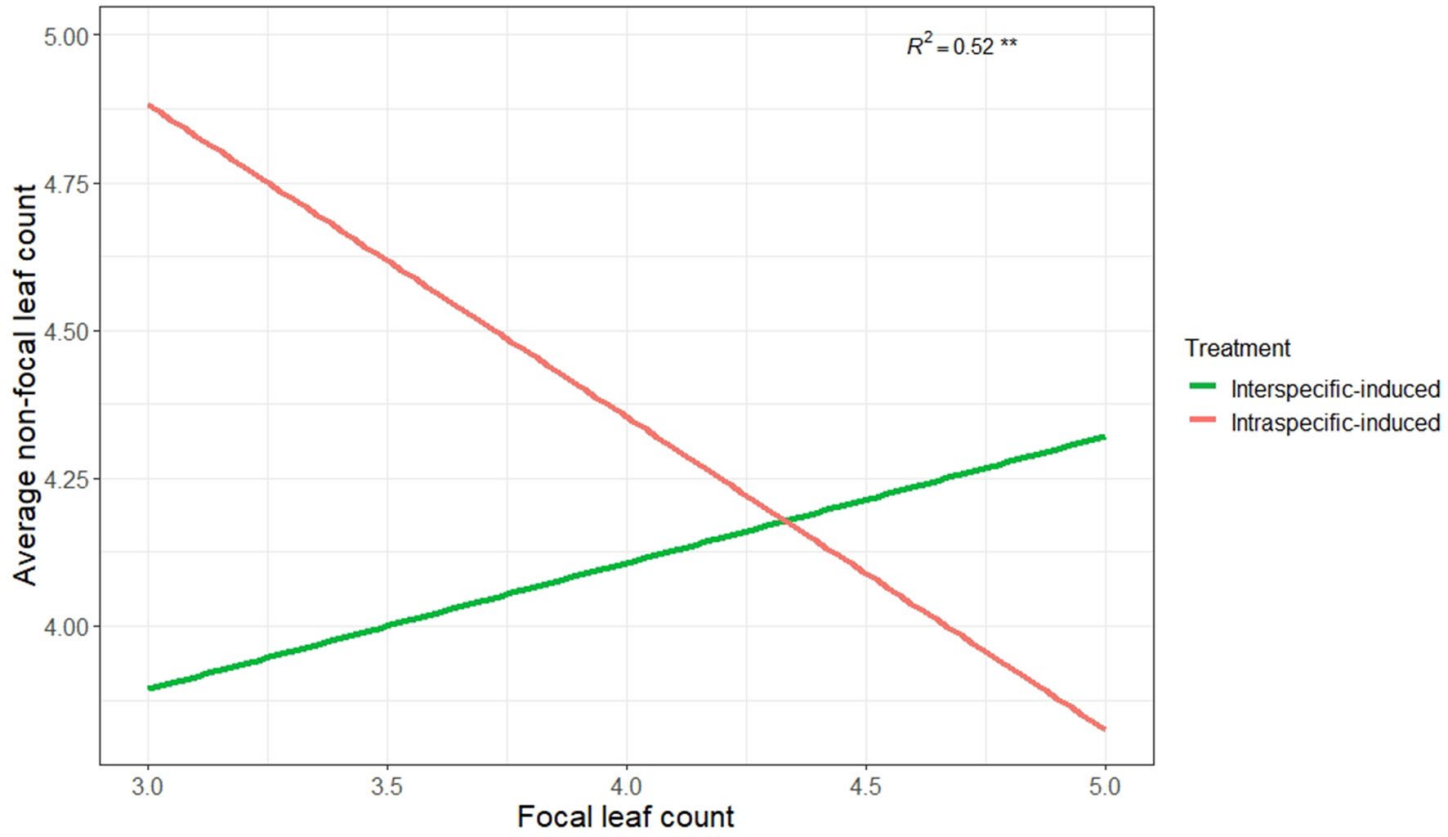
Relationship between *Pascopyrum smithii* focal plant number of leaves and the average number of leaves for the two non-focal conspecific neighbors. Significance levels are denoted by ns *P* > 0.5; *P ≤ 0.05; **P ≤ 0.01; ***P ≤ 0.001.

When intraspecific-induced and interspecific-induced *P. smithii* interacted with new conspecific neighbors, shoot mass and cumulative rhizome count did not differ between the interspecific-induced and intraspecific-induced focal plants and pots, but number of leaves differed between treatments whereby, interspecific-induced plants produced more leaves. Shoot mass (*R*^2^ = 0.03, *F*_1,16_ = 0.5, *p* = 0.5, Fig. 3a), and cumulative rhizome count did not differ between treatments (*R*^2^ = 0.03, *F*_1,16_ = 0.56, *p* = 0.5, Fig. 3b), however the number of leaves differed between the two treatments (*R*^2^ = 0.62, *F*_1,16_ = 0.5, *p* = 0.0001, Fig. 3c). Leaf trait hierarchy was higher in interspecific-induced (x̅ = 0.05) than intraspecific-induced (x̅ = − 0.57) treatments but did not statistically differ between treatments (*R*^2^ = 0.07, *F*_2,14_ = 1.1, *p* = 0.3, data not shown^30^). However, the induced focal plants impact on non-focal plants differed (*R*^2^ = 0.52, *F*_2,14_ = 7.5, *p* = 0.006, Fig. 4) in which the relationship between induced focal plant leaf production negatively affected average number of leaves produced by the non-focal plants in the intraspecific-induced treatments (y = − 0.5x + 5.9), whereas there was a positive relationship in the interspecific induced treatment (y = 0.3x + 2.8).

## Discussion

This study addressed whether interactions induced plastic responses and impact subsequent interactions and shows that interspecific interactions with *Bromus tectorum* induced plastic responses in traits and morphology of *Pascopyrum smithii*, whereas intraspecific interactions lacked plastic responses in many traits. Importantly, intraspecific-induced plants and interspecific-induced differed in their competitive effect upon subsequent interactions with new conspecifics, in which increasing leaf count in the interspecific-induced plants facilitated, and the intraspecific-induced plants suppressed leaf production in the non-focal plants. In empirically demonstrating that different previous plant-plant interactions influence subsequent interactions with intraspecific neighbors, these findings have potential to add to the knowledge base of species coexistence through the effects on subsequent conspecific interactions—an important component of stabilizing niche mechanisms.

Interspecific interactions with *Bromus tectorum* induced plastic responses in *Pascopyrum smithii* plants, as evidenced by increases in traits and biomass responses for root mass, shoot mass, and plant height, but not for root mass fraction (RMF). Positive interactions with *B. tectorum* is surprising given the suppressive impacts of *B. tectorum* on other native grass competitors^13,34^, but similar outcomes have been seen between *P. smithii* and *B. tectorum*^35^. Another interpretation could be of strong competition between these plants in which *P. smithii* increased allocation as a competitive response to reduce potential suppression and performance losses under the presence of *B. tectorum*. Knowing the response of *B. tectorum* would provide more insight into this relationship, but measures on *B. tectorum* were not recorded. Plastic response in plant height to competition may have in-part been due to facilitation or due to the need to improve access to light for photosynthesis, coupled with shade avoidance responses to neighboring *B. tectorum* or acted as a cue indicating the presence of a neighbor^36^*.* Plant height is an important trait that influences light capture for photosynthesis and plays an important role in ecological strategy^37^. Shade avoidance responses can include increases in plant height that aid in access to light and can in-turn impose competition on neighbors^38^, but the responses of *B. tectorum* and non-focal *P. smithii* are unknown in this study.

Traits and morphology following intraspecific interactions, on the other hand, were largely canalized in which root mass and shoot mass did not differ from the control group. RMF and plant height differed from the control indicating plastic responses in these traits. Plastic response in the functional trait, RMF, has ecological significance: RMF can increase strongly to stress^39^, such as water and nutrient stress (a putative stressor in competition) and to competition^6,13,40^. Changes in RMF and plant height are adaptive response as they improve persistence and establishment under the new environment^41^. This may provide benefits in the long-term for interacting *P. smithii* conspecifics under intense competition. Furthermore, for species coexistence, Bennett et al.^42^ showed that stronger intraspecific competition resulted when root allocation was greater and more intense intraspecific competition should directly influence and strengthen stabilizing niche mechanisms^26^.

Root tip count and number of leaves were canalized for both intraspecific and interspecific interactions. This is surprising for root tips because lateral root tip proliferation responds highly plastically to competition and resources stress^43^, for instance, *Lolium perenne* experienced an eightfold increase in lateral roots (a major constituent of root tip count) under drought stress^43^.

In experiment two, leaf count was suppressed in intraspecific-induced relative to the interspecific-induced treatment indicating that differences in performance between the focal plants persisted in interactions with new conspecific neighbors. The previous interaction environment also produced a different relationship between the focal plant and non-focal neighbor’s leaf production indicating that interspecific-induced and intraspecific-induced plants differed in responses to trait hierarchy with the new neighbors. Trait hierarchies are often explored between species^33,42^, however trait differences exist below the species level^34,44,45^ and influence coexistence^46^. For example, Abbott and Stachowicz^46^ showed that increasing root mass distance in pairwise competing genotypes of eelgrass (*Zostera marina*) decreased the probability of genotype coexistence. Generally, the results of the present study suggest that previous intraspecific interactions act to stabilize subsequent interactions. This is achieved by leading to more intense conspecific competition that may result in self-limiting population dynamics that act under a strong source of competition from conspecifics^19,20^. This is corroborated by results from a meta-analysis of phenomenological studies of intraspecific and interspecific interactions^47^. This study presents novel evidence that intraspecific interactions can be weakened by previous interactions with interspecific invasive plants and may play a role in weakening stabilizing niche mechanisms. This invasive plant-mediated change to conspecific interactions may provide some insight into invasive species-mediated population decline if the increasing facilitation results in positive population growth that is not stable under low densities. However, longer term data would be required to address changes to population dynamics. This study originally aimed to also test the impacts of intraspecific-induced and interspecific-induced plants on both new conspecific and new heterospecific neighbors. However, high mortality of the heterospecific plants precluded this assessment. To further understand both intraspecific and interspecific components of stabilizing niche mechanisms and plasticity, future work should also assess effects onto new conspecifics and non-invasive heterospecific plants.

Future work that aims to elucidate the influence of plant-plant induced plastic responses on subsequent interactions should explore study implications in a less controlled environment to assess the influence of environmental heterogeneity, though trait assessments in controlled settings have relevant relationships to performance in the field^48^. This study provides further support to previous research showing that the identity of associates growing with plants prior to out-planting can impact that plant’s performance and interactions when transplanted to a new environment^49^. Plants grown as plugs for commercial purposes are usually grown with many intraspecific neighbors^50^ including maternal plants grown for seed harvesting. Additionally, plastic responses to intraspecific neighbors compared to interspecific neighbors can also be apparent over multiple generations (e.g., transgenerational plasticity)^51^, so these factors should be considered when growing plants for restoration practices to ensure expected plant performance and meeting restoration goals. Lastly, plant life stage can influence the outcomes of species interactions^52^. For instance, the intensity of conspecific competition for the cycad *Dioon sonorense* decreased with age, whereas heterospecific competition increased with age^52^. Seedlings, like the ones used here, are thought to be more sensitive to competition^27,28^, suggesting that the current study on seedlings is informative to plant interaction knowledge base. Transplant studies have been instrumental in elucidating the effects of novel competitors and environments^18^, but excavation can impact plant growth. However, because all treatments were excavated in the same manner, this allows conclusions to be drawn based on the interaction treatments imposed. Future studies should explicitly incorporate plant interactions at different life stages in transplant studies and incorporate multiple interactions to determine consequences of plasticity in subsequent interactions.

## Conclusions

This study provides evidence of how plasticity induced by interactions may affect stabilizing mechanisms by altering intraspecific interactions and has implications for its impact on species coexistence. I show that previous intraspecific interactions may function to further stabilize niche differences, whereas previous interspecific interactions with a non-native invasive species can weaken them, which has implications for better understanding community dynamics of plants that encounter new neighbors in their lifetime. Future research should assess induced plasticity in different abiotic and biotic contexts to provide more empirical evidence for the ecological consequences of plasticity.

## Data availability statement

The datasets generated and R code used to process and analyze these data are available from Mendeley data via: http://dx.doi.org/10.17632/hhpnttctth.1.

## Supplementary Information

Supplementary Figure S1.

## References

[1] Bradshaw AD (1965). Evolutionary significance of phenotypic plasticity in plants. Adv. Genet..

[2] Sultan SE (1995). Phenotypic plasticity and plant adaptation. Acta Bot. Neerl..

[3] Callaway RM, Pennings SC, Richards CL (2003). Phenotypic plasticity and interactions among plants. Ecology.

[4] Fordyce JA (2006). The evolutionary consequences of ecological interactions mediated through phenotypic plasticity. J. Exp. Biol..

[5] Owusu-Nketia S (2018). Functional roles of root plasticity and its contribution to water uptake and dry matter production of CSSLs with the genetic background of KDML105 under soil moisture fluctuation. Plant Prod. Sci..

[6] Acciaresi H, Guiamet J (2010). Below- and above-ground growth and biomass allocation in maize and *Sorghum halepense* in response to soil water competition. Weed Res..

[7] Oduor AMO (2013). Evolutionary responses of native plant species to invasive plants: A review. New Phytol..

[8] Mealor B, Hild AL (2006). Potential selection in native grass populations by exotic invasion. Mol. Ecol..

[9] Ferrero-Serrano Á, Hild AL, Mealor BA (2011). Can invasive species enhance competitive ability and restoration potential in native grass populations?. Restor. Ecol..

[10] Goergen EM, Leger EA, Espeland EK (2011). Native perennial grasses show evolutionary response to *Bromus tectorum* (cheatgrass) invasion. PLoS ONE.

[11] Melgoza G, Nowak RS, Tausch RJ (1990). Soil water exploitation after fire: competition between *Bromus tectorum* (cheatgrass) and two native species. Oecologia.

[12] Reichenberger G, Pyke DA (1990). Impact of early root competition on fitness components of four semiarid species. Oecologia.

[13] Phillips AJ, Leger EA (2015). Plastic responses of native plant root systems to the presence of an invasive annual grass 1. Am. J. Bot..

[14] Cipollini D, Purrington CB, Bergelson J (2003). Costs of induced responses in plants. Basic Appl. Ecol..

[15] Relyea R (2002). Competitor-induced plasticity in tadpoles: consequences, cues, and connections to predator-induced plasticity. Ecol. Monogr..

[16] War AR, Sharma HC, Paulraj MG, War MY, Ignacimuthu S (2011). Herbivore induced plant volatiles: their role in plant defense for pest management. Plant Signal. Behav..

[17] Karban AR, Baldwin IT, Baxter KJ, Laue G, Felton GW (2000). Communication between plants: induced resistance in wild tobacco plants following clipping of neighboring sagebrush. Oeciologia.

[18] Alexander JM, Diez JM, Levine JM (2015). Novel competitors shape species’ responses to climate change. Nature.

[19] Chesson P (2000). Mechanisms of maintenance of species diversity. Annu. Rev. Ecol. Syst..

[20] HilleRisLambers J, Adler PB, Harpole WS, Levine JM, Mayfield MM (2012). Rethinking community assembly through the lens of coexistence theory. Annu. Rev. Ecol. Evol. Syst..

[21] Mayfield MM, Levine JM (2010). Opposing effects of competitive exclusion on the phylogenetic structure of communities. Ecol. Lett..

[22] Muthukrishnan R, Sullivan LL, Shaw AK, Forester JD (2020). Trait plasticity alters the range of possible coexistence conditions in a competition–colonisation trade-off. Ecol. Lett..

[23] Pérez-Ramos IM, Matías L, Gómez-Aparicio L, Godoy Ó (2019). Functional traits and phenotypic plasticity modulate species coexistence across contrasting climatic conditions. Nat. Commun..

[24] Roscher C, Schumacher J, Schmid B, Schulze E-D (2015). Contrasting effects of intraspecific trait variation on trait-based niches and performance of legumes in plant mixtures. PLoS ONE.

[25] Liu B (2015). Complementarity in nutrient foraging strategies of absorptive fine roots and arbuscular mycorrhizal fungi across 14 coexisting subtropical tree species. New Phytol..

[26] Turcotte MM, Levine JM (2016). Phenotypic plasticity and species coexistence. Trends Ecol. Evol..

[27] Foster BL (1999). Establishment, competition and the distribution of native grasses among Michigan old-fields. J. Ecol..

[28] James JJ, Svejcar TJ, Rinella MJ (2011). Demographic processes limiting seedling recruitment in arid grassland restoration. J. Appl. Ecol..

[29] Larson JE, Anacker BL, Wanous S, Funk JL (2020). Ecological strategies begin at germination: Traits, plasticity and survival in the first 4 days of plant life. Funct. Ecol..

[30] Foxx A (2021). Data: Induced plasticity alters responses to conspecific interactions in seedlings of a perennial grass. Mendeley Data.

[31] R Core Team. R: A language and environment for statistical computing. *R Found. Stat. Comput. Vienna, Austria* (2020).

[32] Crawley MJ (2005). Statistics: An introduction using R.

[33] Kraft NJB, Crutsinger GM, Forrestel EJ, Emery NC (2014). Functional trait differences and the outcome of community assembly: An experimental test with vernal pool annual plants. Oikos.

[34] Foxx AJ, Kramer AT (2020). Variation in number of root tips influences survival in competition with an invasive grass. J. Arid Environ..

[35] McGlone CM, Sieg CH, Kolb TE, Nietupsky T (2011). Established native perennial grasses out-compete an invasive annual grass regardless of soil water and nutrient availability. Plant Ecol..

[36] Liu JG, Mahoney KJ, Sikkema PH, Swanton CJ (2009). The importance of light quality in crop-weed competition. Weed Res..

[37] Westoby M (1998). A leaf-height-seed (LHS) plant ecology strategy scheme. Plant Soil.

[38] Gundel PE, Pierik R, Mommer L, Ballaré CL (2014). Competing neighbors: Light perception and root function. Oecologia.

[39] Poorter H (2012). Biomass allocation to leaves, stems and roots: Meta-analyses of interspecific variation and environmental control. New Phytol..

[40] Berendse F, Móller F (2009). Effects of competition on root-shoot allocation in *Plantago lanceolata* L.: Adaptive plasticity or ontogenetic drift?. Plant Ecol..

[41] Ghalambor CK, McKay JK, Carroll SP, Reznick DN (2007). Adaptive versus non-adaptive phenotypic plasticity and the potential for contemporary adaptation in new environments. Funct. Ecol..

[42] Bennett JA, Riibak K, Tamme R, Lewis RJ, Pärtel M (2016). The reciprocal relationship between competition and intraspecific trait variation. J. Ecol..

[43] Jupp A, Newman I (1987). Morphological and anatomical effects of severe drought on the roots of *Lolium perenne* L. New Phytol..

[44] Foxx AJ, Kramer AT (2020). Hidden variation: Cultivars and wild plants differ in trait variation with surprising root trait outcomes. Restor. Ecol..

[45] Zeldin J, Lichtenberger TM, Foxx AJ, Webb Williams E, Kramer AT (2020). Intraspecific functional trait structure of restoration-relevant species: Implications for restoration seed sourcing. J. Appl. Ecol..

[46] Abbott JM, Stachowicz JJ (2016). The relative importance of trait vs genetic differentiation for the outcome of interactions among plant genotypes. Ecology.

[47] Adler PB (2018). Competition and coexistence in plant communities: Intraspecific competition is stronger than interspecific competition. Ecol. Lett..

[48] Schroeder-Georgi T (2016). From pots to plots: Hierarchical trait-based prediction of plant performance in a mesic grassland. J. Ecol..

[49] Taylor D, Aarssen L (1990). Complex competitive relationships among genotypes of three perennial grasses: Implications for species coexistence. Am. Nat..

[50] Espeland EK (2017). Evolution of plant materials for ecological restoration: Insights from the applied and basic literature. J. Appl. Ecol..

[51] Rottstock T, Kummer V, Fischer M, Joshi J (2017). Rapid transgenerational effects in *Knautia arvensis* in response to plant community diversity. J. Ecol..

[52] Álvarez-Yépiz Juan C., Búrquez Alberto, Dovčiak Martin (2014). Ontogenetic shifts in plant–plant interactions in a rare cycad within angiosperm communities. Oecologia.

