# Supplementary material for: Induced plasticity alters responses to conspecific interactions in seedlings of a perennial grass
Source: Sci Rep. 2021 Jul 16;11:14581. doi: 10.1038/s41598-021-93494-0 (PMC8285392; doi:10.1038/s41598-021-93494-0)

Induced plasticity alters responses to conspecific interactions in seedlings of a perennial grass

Plant Ecology

Alicia J. Foxx[1]

[1] Genomics and Bioinformatics Research Unit; United States Department of Agriculture, Agricultural Research

Service, 1600 SW 23rd Drive Gainesville, FL 32608, USA

E: alicia.foxx@usda.gov

Supplementary Information

SI Fig. 1 Transplant procedure following induction from interactions to movement to new pots with new conspecific neighbors

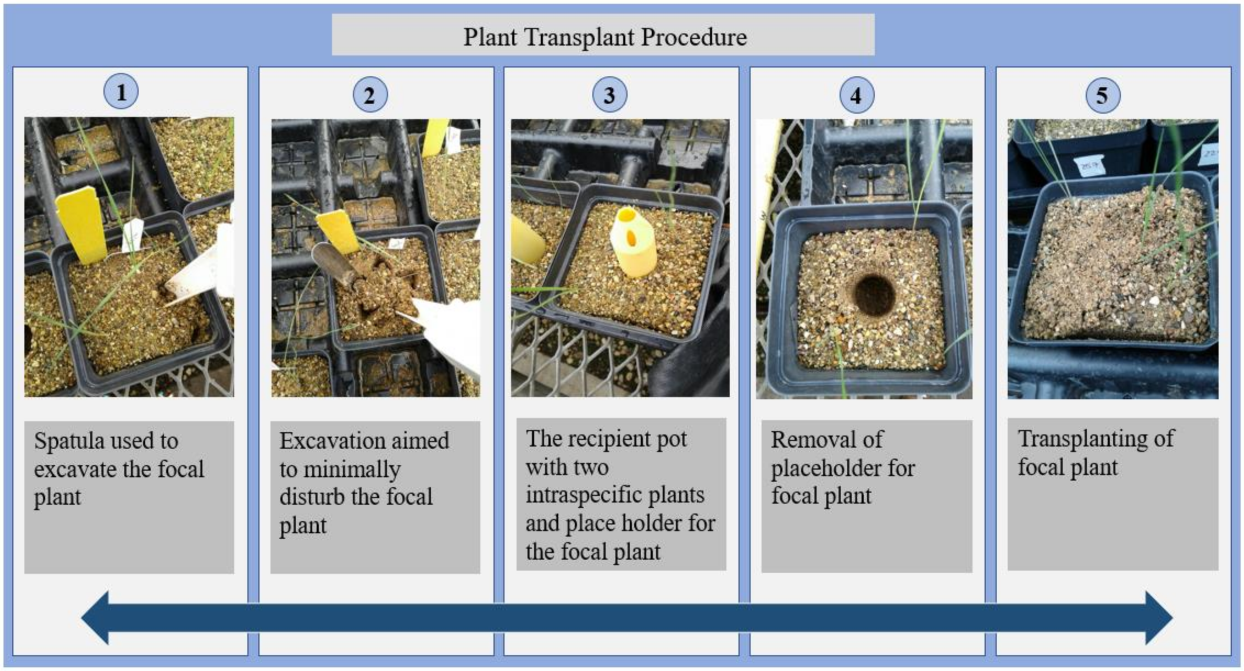

Supplement: Supplementary file 1 — Supplementary Figure S1. [file 41598_2021_93494_MOESM1_ESM.pdf]
